# CD166 expression in dentigerous cyst, keratocystic odontogenic tumor and ameloblastoma

**DOI:** 10.4317/jced.52381

**Published:** 2016-07-01

**Authors:** Azadeh Andisheh-Tadbir, Ali Gorgizadeh

**Affiliations:** 1 Associate Professor. Prevention of Oral and Dental Disease Research Center, Department of Oral and Maxillofacial Pathology, School of Dentistry, Shiraz University of Medical Sciences, Shiraz, Iran; 2 Undergraduate Student. School of Dentistry, Shiraz University of Medical Sciences, Shiraz, Iran

## Abstract

**Background:**

CD166 is a glycoprotein of an immunoglobulin super family of adhesion molecules that has been associated with aggressive characteristics and high recurrence rate of tumors. Different odontogenic lesions exhibit considerable histological variation and different clinical behavior. In an attempt to clarify the mechanisms underlying this different behavior, the present study investigates the immunohistochemical expression of CD166 in these lesions.

**Material and Methods:**

In this study 69 formalin-fixed, paraffin embedded tissue blocks of odontogenic lesion consist of 15 unicystic ameloblastoma (UA), 17 solid ameloblastoma (SA), 18 keratocystic odontogenic tumors (KCOT), and 19 dentigerous cysts (DC) were reviewed by immunohistochemistry for CD166 staining.

**Results:**

In this study, CD166 immune staining was evident in all specimen groups except dentigerous cyst. In positive cases, protein localization was cytoplasmic and/or membranous. CD166 expression was seen in76.5% (13) of SA, 73.5% (11) of UA, and 66.7% (12) of KCOTs. Statistical analysis showed that CD166 expression levels were significantly higher in ameloblastoma (SA and UA) and KCOTs than dentigerous cyst (*P* <0.001), but there was no statistically significant difference between CD166 expression in the other groups (*P*>0.05).

**Conclusions:**

This data demonstrates that overexpression of CD166 may have a role in the pathogenesis of ameloblastoma and KCOT.

** Key words:**CD166, ameloblastoma, dentigerous cyst, odontogenic keratocyst.

## Introduction

Cell junctions connect epithelial cells to one another and consist of various proteins, generically called adhesion molecules, which are keys to the regulation of cell growth and differentiation (1).

Adhesion molecules are involved in tumor cell-tumor cell adhesion, tumor cell-endothelial cell adhesion, and tumor cell-matrix adhesion, all of which are necessary for primary tumor formation or metastasis (2).

A variety of molecules that regulate cell adhesion have been identified and investigated. Adhesion molecules are divided into five major groups: immunoglobulins, cadherins, selectins, integrins and mucins (2).

Activated leukocyte cell adhesion molecule, or CD166, is a glycoprotein of an immunoglobulin superfamily of adhesion molecules (IgCAMs). IgCAMs are mostly transmembrane proteins that can function as cell adhesion receptors and transduce signals to intracellular signaling pathways (3). The CD166 gene is located on chromosome 3 and is composed of 16 exons with a size of over 200 Kb (4).

CD166 is involved in the development of different tissues during embryogenesis and functions in adults through homotypic and heterotypic interactions between cells (4-6). The expression of CD166 has been discovered in a subgroup of cells which have a role in dynamic growth and migration. It has, however, been discovered in cancer stem cells as well (6).

Altered CD166 expression has also been associated with differentiation and progression in prostate (7,8), colorectal (9), and breast cancers (10).

CD166 expression reflects aggressive characteristics and can be associated with high recurrence rate of tumors (9,11).

Different odontogenic lesions arise from the element of the tooth-forming apparatus, and its derivatives exhibit considerable histological variation and different clinical behavior (12,13).

Dentigerous cyst is the most common developmental odontogenic cyst with an indolent behavior and a low recurrence rate (14).

Keratocystic odontogenic tumor (KCOT) has recently been recommended to describe the lesion previously named odontogenic keratocyst (15) KCOT is a developmental cyst which is locally aggressive and rapidly proliferating and shows a high recurrence rate after removal (16).

Since radical procedures are suggested for KCOT in contrast to dentigerous cyst, the histological investigation of this entity has been the focus of many studies (17).

Ameloblastoma is a benign odontogenic tumor with an aggressive behavior and a marked invasive potential that leads to multiple recurrences after enucleating and curettage (18).

Previous studies disclose the role of different adhesion molecules in the pathogenesis and aggressive behavior of odontogenic lesions (19,20).

In view of the distinct clinical behavior of KCOT, dentigerous cyst, and ameloblastoma, the objective of the present study was to investigate the immunohistochemical expression of CD166 in these lesions.

## Material and Methods

In this study 69 formalin-fixed, paraffin embedded tissue blocks of odontogenic lesion consist of 15 unicystic ameloblastoma (UA), 17 solid ameloblastoma (SA), 18 keratocystic odontogenic tumors (KCOT), and 19 dentigerous cysts (DC) were reviewed by immunohistochemistry for CD166 staining.

This study was approved by the Ethics Committee of the Shiraz University of Medical Sciences.

H & E slides of available blocks were reviewed, and then cases with definite diagnosis and adequate tissue were selected for immunohistochemical staining (IHC). Cases with severe inflammation were excluded from the study.

IHC staining was performed using the Envision Labeled Peroxides System (DAKO, Carpentaria, CA, USA). All samples were fixed in 10% buffered formalin and embedded in paraffin. Sections of 4μ thickness were prepared and deparaffinized in histology grade Xylene, rehydrated in graded alcohol, and washed with distilled water. Antigen retrieval was performed using DAKO cytomation target retrieval solution with PH = 9 for 20 minutes. Internal peroxidase activity was inhibited by 3% H2O2.

Tissue sections were incubated for 30 minutes with the anti-CD166 antibody (clone: MOG/07, 1:450; Novocastra, UK) at 1/100. Normal samples were stained with the same amount of antibody used for staining tumor tissues. Omission of primary antibody was employed as the negative control, while oral squamous cell carcinoma was used as the positive control.

Brown staining of the cell membrane, cytoplasm or both was considered as positive. The immunopositive staining was evaluated in 5 randomly-selected areas of the tissue in the basal and parabasal layers of cystic lesions and peripheral cells of ameloblastic nests (magnification was 400×), and the percentage of positive tumor cells was calculated.

A lesion was designated as overall CD166 positive if greater than 10 % of cells exhibited immunoreactivity.

Chi squareand Fisher’s exact tests were used to compare results. A *p* value less than 0.05 was considered significant.

## Results

**Figure 1:**
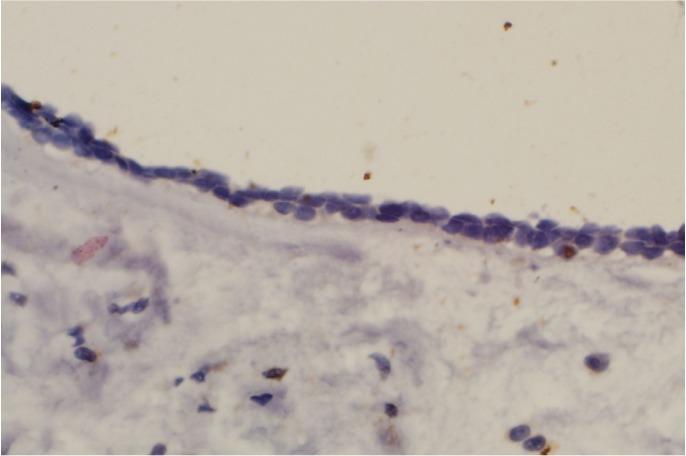
Negative CD166 expression in dentigerous cyst (×400).

In this study, CD166 immune staining was evident in all specimen groups except dentigerous cyst (Fig. 1). In positive cases, protein localization was cytoplasmic and/or membranous. CD166 expression was seen in 76.5% (13) of SA, 73.5% (11) of UA, and 66.7% (12) of KCOTs. Statistical analysis showed that CD166 expression levels were significantly higher in ameloblastoma (SA and UA) and KCOTs than dentigerous cyst (*P*<0.001), but there was no statistically significant difference between CD166 expression in the other groups (*P*>0.05), ( Table 1).

**Table 1:**
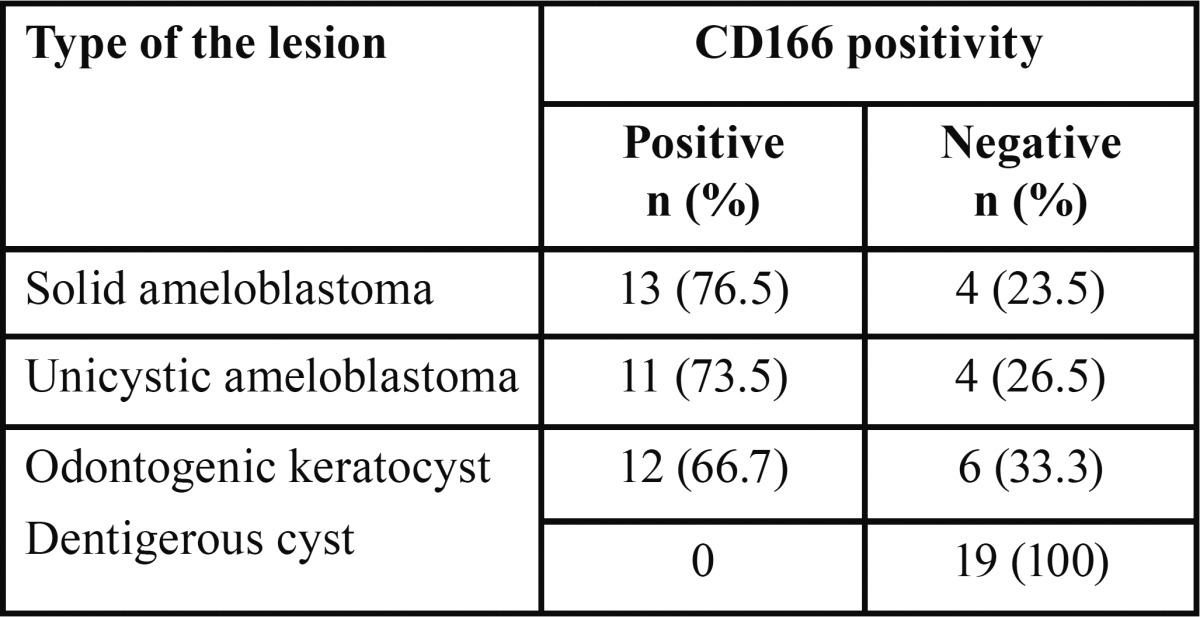
CD166 expression in different groups of lesion.

**Figure 2:**
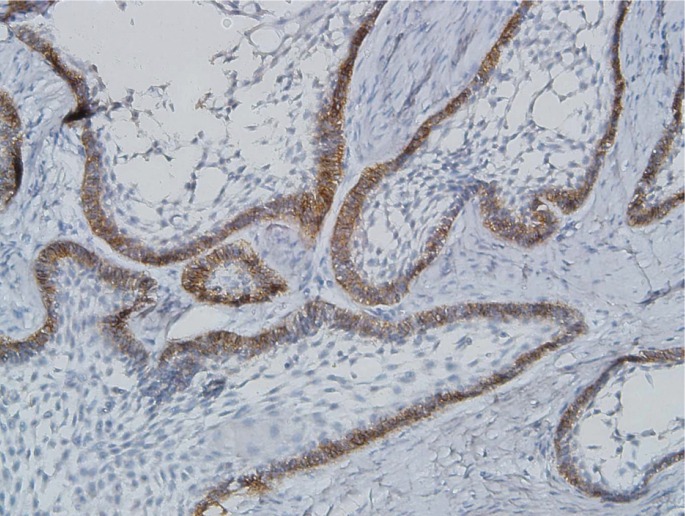
Cytoplasmic/membranus CD166 expression in ameloblast like cell in solid ameloblastoma (×200).

**Figure 3:**
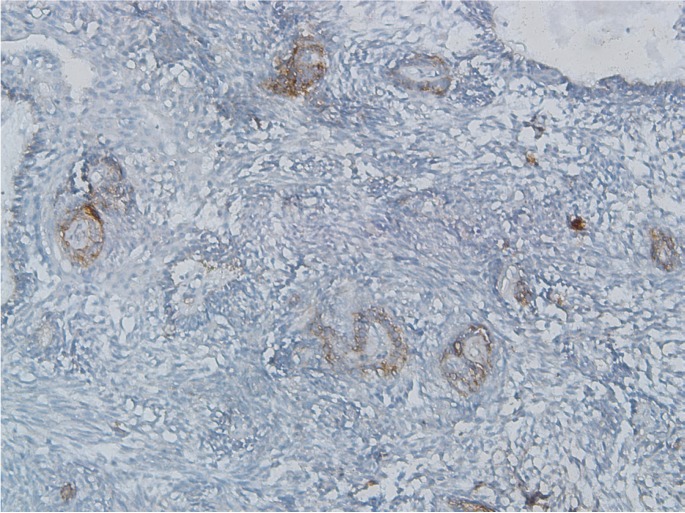
Cytoplasmic/membranus CD166 expression in plexiform ameloblastoma (×200).

Expression of CD166 in follicular and plexiform SAs was the same. CD166 positivity was observed on the cytoplasm and/or cell membrane of peripheral columnar cells and was negative in central stellate reticulum-like cells. Expression of the molecule was lost in the keratinized areas of acanthomatous SAs (Figs. 2,3).

**Figure 4:**
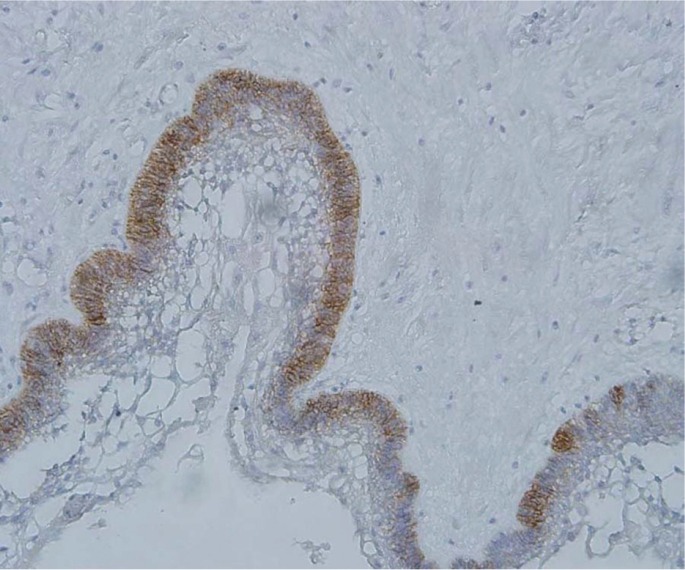
Cytoplasmic/membranus CD166 expression in unicystic ameloblastoma (×200).

The UAs showed a reaction in the basal layer of the cystic lining, but no reaction in the upper layers (Fig. 4).

**Figure 5:**
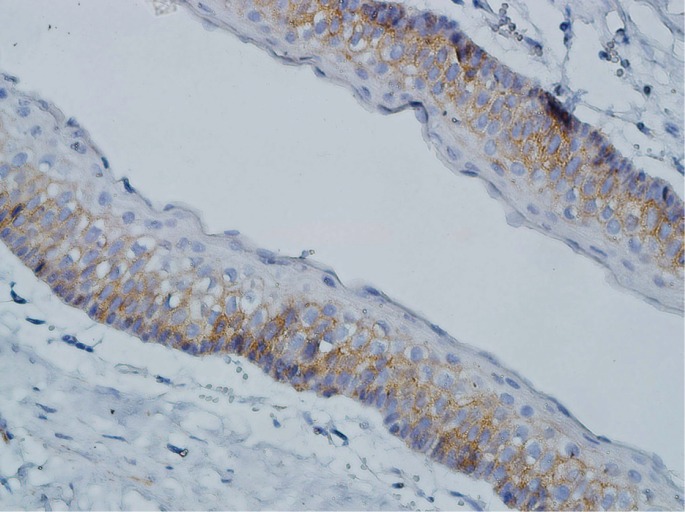
Cytoplasmic/membranus CD166 expression in odontogenic keratocyst (×400).

Expression of CD166 in KCOTs was positive in the cell membrane and/or cytoplasm of most of the cells in the basal and supra-basal layers, and all layers of epithelial budding and daughter cysts (Fig. 5).

## Discussion

The invasion, which means the penetration of tumor cells into the adjacent environment, is not specific to cancer; it is also found in embryogenesis, healthy organs, and in many non-cancerous diseases (21).

The process of invasion is exceedingly complex, and the genetic and biochemical determinants are still unclear (22). Recent studies have found genetic and molecular alterations in epithelial odontogenic tumors; however, details of the mechanism of oncogenesis, cytodifferentiation, and tumor progression remain unknown (23).

Recent studies have reported that the expression of CD166 markers in tumors indicates the aggressive behavior of the malignancies *in vitro* (5,24). The present study is the first to have demonstrated the expression of CD166 in odontogenic lesions.

In the present study, CD166 immunoreactivity was higher in ameloblastomas and KCOTs than in DCs. This result indicates that CD166 may play a role in the pathogenesis of ameloblastomas and KCOTs but not in DC.

The difference in CD166 expression observed in the present study might explain the variable behavior of these lesions, the highly aggressive and invasive behavior of ameloblastomas and KCOTs, and it supports the concept that KCOT has a neoplastic potential.

There was no difference in CD166 expression with respect to unicystic and solid ameloblastoma. It is possible that the differences in the clinical behavior of both tumors are determined by other factors than those directly associated with the expression of this molecule.

Two different cell types are observed in all subtypes of ameloblastoma: peripheral columnar or ameloblast-like cells and central stellate reticulum-like cells (25). Peripheral cells are always located at the invasive front, but show no cellular differentiation. By contrast, in many cases morphological differentiation was observed in central stellate reticulum-like cells (25).

The present experiment found that positive CD166 immunohistochemical reactivity was prominent on the peripheral columnar cells and absent on the stellate reticulum-like central cells. This result supports the thesis that these cells are located at the invasive front, and overexpression of CD166 at these cells may promote invasion. However, this particular localization of CD166 expression close to the stroma might emphasize other role of CD166 such as cancer stem cell or raise the question of whether factors secreted from stromal cells might induce CD166 expression.

A previous study demonstrated that CD166 is a TGF-B responsive marker (26). This finding supports the possibility that stromal growth factors may enhance CD166 expression and contribute to regulation of the invasion of ameloblastoma.

Kaur *et al.* demonstrated that loss of membrane E-cadherin correlated significantly with overall CD166 immunopositivity in hyperplasia of the oral cavity (27).

Gonzalez-Alva *et al.* showed that expression of E-cadherin decreased in areas of cell-cell contact of the peripheral cells in AMs and retained E-cadherin in the central areas of tumor cells (28) and Hakim *et al.* found that E-cadherin staining decreased in KCOTs compared with dentigerous cyst (15). To clarify the correlation of these two proteins in the odontogenic lesions future studies are recommended.

Different cell behaviors are involved in the formation of epithelial budding and daughter cyst compared with cyst formation. Proliferation of basal cells with the accumulationof cyst contents lead to the wall pushing outward and cyst formation. In contrast, invasive protrusion of KCOT nests into the adjacent stromal tissue results in epithelial islands and daughter cyst formation, which is reminiscent of the early stages of tooth germ formation (29).

Shimeda *et al.* proposed that these histological features may represent the invasive potential of KCOT (29).

In the present study, positive immunoreactivity for CD166 was also found in all layers of daughter cyst and epithelial budding, which supports the hypothesis of Shimeda *et al.* (29) and the invasive potential of KCOT.

The mechanism by which CD166 expression contributes to tumor progression is currently unclear (30). However, Lunter *et al.* recently found that the proper function of the matrix metalloproteinase-2 (MMP-2) was dependent on CD166 expression and its function (31).

This finding suggests that CD166 has a signaling role in proteolysis, and CD166 overexpression can enhance MMP-2 activity and the breakdown of the extracellular matrix, thus resulting in increased tumor invasiveness and progression (31).

Khalifa *et al.* found that overexpression of the MMP-2 protein may be one of the factors related to the growth and progression of KCOT and ameloblastoma, and it may enhance the recurrence of KCOT and invasion of ameloblastoma (32).

Because of the unknown biological role of CD166, future *in-vitro* studies should focus on the physiological and pathological molecular mechanisms and interactions of these molecules.

In conclusion, in this study overexpression of CD166 was seen in ameloblastoma and KCOT which might be related to the aggressive behavior and high recurrence rate of these tumors but for supporting this hypothesis future studies are recommended.
